# Updating the Free Radical Theory of Aging

**DOI:** 10.3389/fcell.2020.575645

**Published:** 2020-09-16

**Authors:** Adam S. Ziada, Marie-Soleil R. Smith, Hélène C. F. Côté

**Affiliations:** ^1^Department of Pathology and Laboratory Medicine, University of British Columbia, Vancouver, BC, Canada; ^2^Centre for Blood Research, University of British Columbia, Vancouver, BC, Canada; ^3^Women's Health Research Institute, Vancouver, BC, Canada

**Keywords:** free radical theory of aging, mtDNA, mtDNA mutations, oxidative stress, clonal expansion, polymerase gamma (POLG), mtDNA replication, aging

## Introduction

The free radical theory of aging, one of the nine suggested hallmarks of aging (López-Otín et al., 2016), implicates the gradual accumulation of oxidative cellular damage as a fundamental driver of cellular aging (Harman, 1956; Miquel et al., 1980). This theory has evolved over time to emphasize the role of free radical induced mitochondrial DNA (mtDNA) mutations and the accumulation of mtDNA deletions (Miquel et al., 1980; Cortopassi et al., 1992; Michikawa et al., 1999). Given the proximity of mtDNA to the electron transport chain, a primary producer of free radicals, it postulates that the mutations would promote mitochondrial dysfunction and concomitantly increase free radical production in a positive feedback loop. The observation of oxidative damage in the form of 7,8-dihydro-8-oxo-deoxyguanosine (8-oxodG) DNA oxidative lesions accumulating with age has been a cornerstone of the free radical theory of aging (Fraga et al., 1990).

## The Influence of Stressors On mtDNA Mutation Burden and Aging

A major assumption of the free radical theory of aging is that random *de novo* or somatic mtDNA mutations gradually accumulate over time, eventually reaching pathological levels (Harman, 1956, 1972). However, data from Payne et al. support the hypothesis that, rather than gradually accumulating over time, mtDNA turnover can lead to the clonal expansion of pre-existing age-related mutations (Payne et al., 2011). Once amplified, these higher frequency mtDNA mutations, that are potentially pathogenic, are referred to as heteroplasmy.

To further understand the potential link between mtDNA mutations and the free radical theory of aging, our group examined aging in the context of tobacco smoking and human immunodeficiency virus (HIV) infection, both believed to accelerate aging. While smoking has long been known for its association with accelerated aging and oxidative damage (Kiyosawa et al., 1990; Loft et al., 1992), HIV infection is also increasingly studied as a promoter of accelerated aging (Effros et al., 2008; Deeks and Phillips, 2009). HIV-positive individuals experience a reported decrease in lifespan of up to 10 years (Lohse et al., 2007; Antiretroviral Therapy Cohort Collaboration, 2008), as well as earlier onset and higher prevalence of age-related comorbidities (Guaraldi et al., 2011). These include cardiovascular disease, hypertension, diabetes, bone disease, and renal failure among others, even in individuals whose viremia is controlled by antiretroviral therapy (Guaraldi et al., 2011).

**Figure 1:**
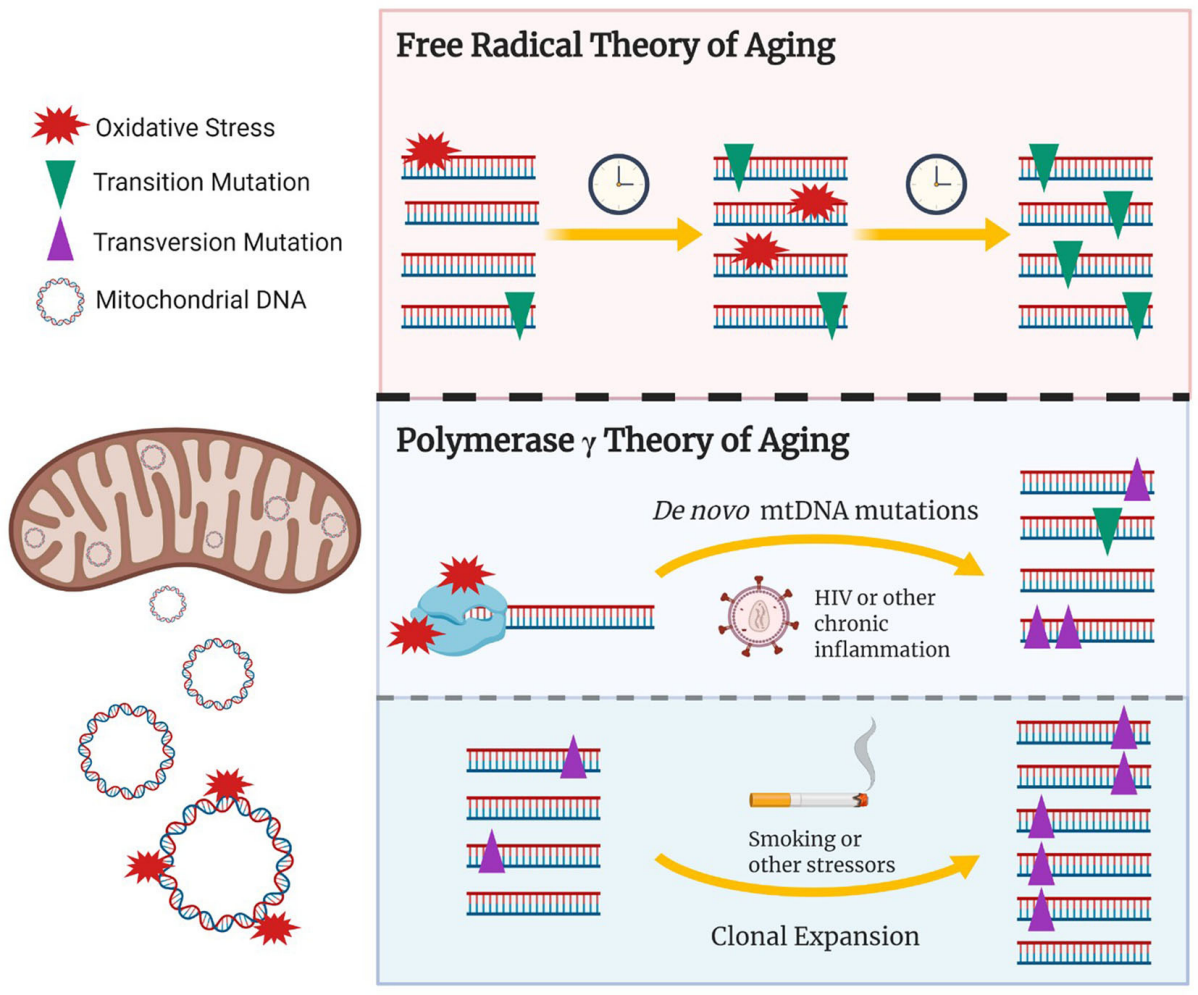
The updated role of oxidative damage and mtDNA polymerase γ in age-associated mtDNA mutations. The free radical theory of aging hypothesizes that oxidative damage to the mtDNA induces random *de novo* mtDNA mutations which gradually accumulate over time, potentially reaching pathological levels. Recent studies have shown that transition mtDNA mutations (purple triangles) rather than transversion mutations (green inverted triangles) gradually build up overtime and are amplified, *via* clonal expansion, to pathological levels. Given that transition mutations are generally associated with replication errors made by the mitochondrial polymerase γ, the age associated accumulation of mtDNA mutations could result from free radicals interacting with polymerase γ, potentially reducing its fidelity and/or inhibiting mtDNA replication. This would in turn lead to random *de novo* transition mutations and their subsequent clonal amplification. Conditions hypothesized to induce accelerated aging *via* oxidative damage/stress could include chronic infections such as HIV, chronic inflammatory conditions, or tobacco smoking. Stressors that induce mitochondrial biogenesis or cellular turnover, which could be mediated *via* oxidative stress, would in turn promote clonal expansion of existing damage. Created with BioRender.com.

Our study found that both random somatic and heteroplasmic mtDNA mutations, the latter defined as a frequency >2%, were associated with older age (Ziada et al., 2019). Further, our data suggest that smoking and HIV may distinctly contribute to the accumulation of mtDNA mutations. Indeed, smoking showed an association with increased mtDNA heteroplasmy but not somatic mutations, while the reverse was observed with HIV participants, but only in those with a history of high viremia, reflecting poor control of HIV. These results suggest that the chronic immune activation and subsequent oxidative stress induced by HIV may lead to *de novo* mtDNA mutations, while oxidative damage associated with exposure to tobacco smoking may promote the clonal amplification of pre-existing mtDNA mutations (Figure 1).

Consistent with this model, no heteroplasmic transversion mutations, typically a signature of oxidative damage, were observed among the 164 participants studied (Ziada et al., 2019). Furthermore, within a given participant showing heteroplasmy, identically mutated molecules of mtDNA would be repeatedly observed; however, between participants, the pattern of heteroplasmic mutation was generally unique (Ziada et al., 2019). Such a pattern is not consistent with the gradual build-up of random mtDNA mutations. Taken together, our findings do not support the slow accumulation of mtDNA transversion mutations as proposed by the free radical theory of aging. Rather, they suggest that randomly mutated molecules of mtDNA are being clonally amplified to generate unique patterns of heteroplasmy in our participants.

## mtDNA Damage and The Role of Polymerase γ

Although the accumulation of mtDNA mutations has been linked to older age and age-associated conditions (Michikawa et al., 1999), several studies have provided new insight that challenge the connection between oxidative damage and mtDNA mutations. For example, the most studied oxidative lesion, 8-oxodG (Yasui et al., 2014), is one of the 37 major oxidative lesions, and is known to induce transversion mutations (A↔ C, A ↔ T, C ↔ G, G ↔ T) (Evans et al., 2004). However, recent studies showing the accumulation of mtDNA mutations with aging did not observe increases in mtDNA transversion mutations, but rather increases in mtDNA transition mutations (A↔ G, C↔ T) (Trifunovic et al., 2004; Kennedy et al., 2013), believed to be the hallmark of mitochondrial polymerase γ errors rather than oxidative damage (Spelbrink et al., 2000; Longley et al., 2001; Kauppila et al., 2017). Additionally, in our study, although both somatic transition and transversion mutations increased with older age, transition mutations were over 30 times more abundant than transversion mutations, once again suggesting that mtDNA replication errors are the major contributors to mtDNA mutation burden (Ziada et al., 2019).

Mutations in mitochondrial polymerase γ, responsible for mtDNA replication in mammalian cells (Hübscher et al., 1979), can be broadly pathogenic and reduce fidelity, resulting in mtDNA replication errors. Over time these errors, including mtDNA point mutations, may undergo clonal expansion, reaching pathogenic levels within the organisms lifetime. Among the first to highlight the importance of polymerase γ errors was a study performed by Trifunovic et al. using mutator mice with proof-reading-deficient mitochondrial polymerase γ. These mice not only showed an increase in mtDNA mutation burden, the vast majority of which were transition mutations, they also displayed a reduced lifespan and the premature onset of aging-related phenotype with no evidence of increased oxidative stress (Trifunovic et al., 2004). This paper provided a causative link between the buildup of polymerase induced mtDNA mutations and aging, forming the foundation for a new polymerase γ focused theory of mitochondrial aging (Trifunovic et al., 2004; Matkarimov and Saparbaev, 2020).

The development of the polymerase γ theory of aging led to renewed examination of the links between free radicals and mitochondrial aging. Mirroring the results in humans (Kennedy et al., 2013; Ziada et al., 2019), a recent study in *Drosophila* showed that age was associated with accumulation of somatic mtDNA transition, but not transversion, mutations suggesting that the role of polymerase γ errors in mitochondrial aging is not limited to humans (Itsara et al., 2014). Building on these results, neither the loss-of-function of antioxidant or DNA damage repair enzymes were shown to increase somatic mtDNA point mutation burden in that model (Itsara et al., 2014). Taken together, these findings lend strong support to the role of polymerase γ errors, with respect to their direct contribution toward somatic mtDNA point mutation burden, and confine free radicals to a comparatively minor role.

Understanding the role that free radicals may play in a polymerase γ centric model of mitochondrial aging is an active area of research. Zsurka et al. suggest that oxidative stress may induce mtDNA deletions rather than point mutations, and that the polymerase γ nucleotide selectivity may prevent the fixation of 8-oxodG induced transversion mutations (Zsurka et al., 2018). Nevertheless, while the 8-oxodG lesion may not directly induce lasting transversion mutations (Basu et al., 1989; Kreutzer and Essigmann, 1998), one or more of the other 37 products of oxidative damage (Evans et al., 2004), some of which induce transition mutations, may contribute to the somatic mtDNA transition mutation burden observed with older age (Kennedy et al., 2013; Ziada et al., 2019).

It has also been suggested that under specific circumstances, reactive oxygen species (ROS) may play an important role in normal mitochondrial function. One *Drosophila* study demonstrated that while ROS production increases with age and correlates with the functional deterioration of mitochondria, increasing ROS production at a choice site within the electron transport chain can act as a signal to maintain mitochondrial function and extend lifespan (Scialò et al., 2016). Studies in *C. elegans* have also suggested that low levels of mitochondrial stress may be protective and extend longevity (Palikaras et al., 2015; Merkwirth et al., 2016). These findings fit into our growing understanding of the role ROS may play in mitochondrial aging, whereby low ROS levels they may be beneficial, but in excess contribute to mitochondrial dysfunction. Through their interaction with mitochondrial polymerase γ, excess of ROS could potentially reduce the enzyme's fidelity, indirectly contributing to an age-associated increase in somatic mtDNA mutations.

An alternate model proposed by Matkarimov and Saparbaev suggest that the spontaneous decay of mtDNA, rather than the accumulation of polymerase γ errors, could be a major source of endogenous mutations (Matkarimov and Saparbaev, 2020). This model is predicated on the spontaneous decomposition of DNA bases (Lindahl and Andersson, 1972; Lindahl and Karlstrom, 1973; Lindahl and Nyberg, 1974), a process that is accelerated when the DNA is in a single stranded form, such as during mtDNA replication, and would result in the accumulation of transition mutations over time (Matkarimov and Saparbaev, 2020). While this model very effectively explains the accumulation of somatic transversion mutations with older age (Kennedy et al., 2013; Ziada et al., 2019), mechanism(s) have been put forward by which such decay could be affected by factors and diseases with increased somatic transition mtDNA mutations (Ju et al., 2014; Ziada et al., 2019; Matkarimov and Saparbaev, 2020).

## Conclusion

Recent research support the theory that mtDNA replication errors are the major drivers of cellular mtDNA mutation burden (Trifunovic et al., 2004; Kennedy et al., 2013; Kauppila et al., 2017). Nonetheless they do not exclude a comparatively minor role for 8-oxodG-induced transversion mutations, or the many other DNA oxidative lesions that can induce transition mutations (Basu et al., 1989; Kreutzer and Essigmann, 1998). Based on recent findings, an updated understanding regarding the role of free radicals in contemporary theories of mtDNA aging is needed. It seems likely that rather than directly contributing to mtDNA mutations *via* oxidative lesions, free radicals may affect the mitochondrial polymerase and decrease its fidelity, indirectly increasing somatic transition mutations. ROS may also act as a signaling molecule and influence mitochondrial biogenesis and/or mitochondrial turnover, which could in turn promote the clonal expansion of pre-existing mtDNA mutations.

## Author Contributions

AZ, M-SS, and HC conceived, designed, and wrote the manuscript. All authors contributed to the article and approved the submitted version.

## Conflict of Interest

The authors declare that the research was conducted in the absence of any commercial or financial relationships that could be construed as a potential conflict of interest.
